# Prevalence and risk factors for pelvic organ prolapse in Kilimanjaro, Tanzania: A population based study in Tanzanian rural community

**DOI:** 10.1371/journal.pone.0195910

**Published:** 2018-04-25

**Authors:** Gileard G. Masenga, Benjamin C. Shayo, Vibeke Rasch

**Affiliations:** 1 Kilimanjaro Christian Medical University College, Moshi, Kilimanjaro, Tanzania; 2 Department of Obstetrics and Gynecology, Kilimanjaro Christian Medical Centre, Moshi, Kilimanjaro, Tanzania; 3 Department of Obstetrics and Gynecology, Odense University Hospital, Odense, Denmark; 4 Department of Clinical Research, University of Southern Denmark, Odense, Denmark; Univesity of Iowa, UNITED STATES

## Abstract

**Introduction:**

The prevalence and risk-factors of pelvic organ prolapse (POP) in Tanzania are unknown. To help elucidate the problem, we assessed POP and associated risk-factors among Tanzanian women by deploying the POP-Q classification system.

**Method:**

A cross sectional community based study conducted in Hai, Rombo and Same Districts, Kilimanjaro Region, Tanzania. Women aged 18–90 were recruited through multi-stage random sampling from January to May 2015. Home-based questionnaire interviews were performed and the women were subsequently invited to the nearest health clinic for pelvic examination. Trained physicians used the POP-Q classification system to assess the POP stage.

**Results:**

A total of 1195 women were interviewed and invited for pelvic examination; 1063(89%) women presented at the clinic of whom 1047(88%) accepted a clinical examination. Of 1047 examined women, 64.6% had an anatomical POP stage II–IV and 6.7% had a severe POP that descended 1 cm or more below the hymen. POP stage II–IV was associated with being aged 35+ years, being a farmer, doing petty trading and having delivered 3 times or more. Severe POP was associated with carrying heavy objects for ≥ 5 hours (OR 4.70;1.67–13.2), having delivered 5 times or more (OR 10.2;2.22–48.6) and having delivered at home (OR 2.40;1.36–4.22).

**Conclusion:**

POP is a common condition among rural Tanzanian women where 64.6% are having POP grade II-IV and 6.7% are having a severe POP descending 1 cm or more below the hymen. Risk-factors are increasing age, heavy lifting, high parity and home-delivery.

## Introduction

Pelvic organ prolapse (POP) is considered to be a major cause of morbidity among women in both high-income and low-income countries [1]. The worldwide prevalence of POP has recently been reported to be around 9% [2]. The figure is, however, estimated to be closer to 20% in low-income countries [3]. In sub-Saharan Africa, studies from Ghana, Gambia and Ethiopia have reported prevalence rates which vary from 12–55% [4–6] with the most recent study revealing a 1% prevalence of symptomatic POP in women of reproductive age in Ethopia [7]. This large variation probably reflects methodological challenges in measuring POP.

Globally, the most predominant risk factor for POP is increasing parity [8]. Other well-known risk factors are increasing age, prolonged labour, lifting of heavy objects and obesity [9–12]. POP is common in high-income countries; however, the problem is expected to be even worse in low-income countries, since women in such settings are more prone to early childbirth, many vaginal deliveries and involvement in occupations with heavy lifting [6].

Women who are affected by POP are bothered by a protruding mass in the vagina and report difficulty in sitting, walking, and lifting (89%). In low-income settings, this may affect the women’s acceptance as full family and community members [13]. The social consequences of POP may be substantial, and include physical and emotional isolation [13]. Due to the stigma surrounding pelvic floor disorders, which is pronounced in low-income countries, women affected by POP often hide their situation and do not seek help.

In Tanzania, facility delivery is limited and only 50% of women deliver in a hospital [14]. Very little is known about the prevalence of POP and POP risk factors. Acknowledging the lack of information on POP in Tanzania as well as the methodological challenges in measuring POP, we decided to examine the prevalence and risk factors of POP among Tanzanian women by deploying the POP-Q classification system.

## Materials and methods

### Study setting

This study is part of the Pelvic Floor Disorders in Tanzania (PEDITA) project, which is a collaborative partnership between Kilimanjaro Christian Medical Center (KCMC) in Tanzania and Odense University Hospital and the University of Southern Denmark in Denmark. The study was conducted in Kilimanjaro region in north-eastern Tanzania. The region has a population of 1 640 087 inhabitants of whom 846 987 are females, according to the Tanzania population census of 2012. Administratively, the region is divided into 6 districts, 30 divisions, 60 wards, 153 villages and 472 sub villages.

### Study population

The women were recruited through a multi-stage randomization process between January and May, 2015. Three districts (Hai, Same and Rombo) were chosen by randomly picking three out of six chits with the names of the six districts in Kilimanjaro Region. Using the same simple randomization method, four wards were chosen from each district, then five villages from each of the wards and then four sub villages from each of the villages. With the help of local authorities, 20 households were chosen by systematic sampling, that is, selecting every n^th^ household from the household registers in the village offices. The female head of each of these households were interviewed the next day at their homes by trained study nurses. The n^th^ household was obtained by dividing the total number of households in the registers by 20, which was the number of required households in each village. If there was no female in the household or no one who was eligible for the study, replacement was done by moving to the next household on the list.

### Questionnaires

A face-to-face interview was conducted in the women’s homes using a questionnaire to gather socio-demographic and reproductive information and symptoms of POP and urinary incontinence. Women who were 18 years of age or above, not pregnant at the time of interview and able to give consent, were invited to participate in the study. Symptoms of POP were assessed by two questions adopted from the American RRISK study [8]: 1) Do you have a feeling of bulging/pressure or something seems to be coming out of the vagina? 2) Do you have a visible mass protruding via the vagina? If a woman answered ‘yes’ to one or both of these questions, she was considered as having symptoms of POP.

All interviewed women were given appointments to attend a selected nearby health clinic for clinical examination. At the clinic, a Kiswahili translation of the Pelvic Organ Prolapse Distress Inventory 6 (POPDI-6) and the Pelvic Floor Impact Questionnaire (PFIQ) were used to evaluate POP symptoms and quality of life, respectively [15]. The tools were modified after being translated into Kiswahili and pilot tested on a group of nurses. The questions, ‘Ever have to push on the vagina or around the rectum to have or complete bowel movement?’ in the POPDI-6 and ‘Feeling frustrated?’ in the POPIQ-7 were omitted after failing to be translated into meaningful and easily comprehensible Swahili phrases. These adapted questionnaires were administered by a trained nurse before clinical examination.

### Clinical examination

A trained study nurse measured the heights and weights of the participants. The POP-Q classification system was used in evaluating and staging of POP [16, 17]. The POP-Q examination was performed by a resident trained in the skills together with two gynaecologists. Pelvic examination was done after a woman had emptied her bladder and the procedure had been explained to her. Examination was performed in lithotomy position in an examination bed using Sims speculum. In accordance with the standard POP-Q examination, a graduated wooden tongue depressor was used to measure in centimeters the standard reference points and their distance from the hymen. The point descent in relation to the hymen while performing Valsalva or cough was recorded as the stage in the three areas examined (anterior, posterior and apical/cervix) and the final stage was the maximum one from the three measurements according to the POP-Q classification system (Table 1). In addition, POP with the most distal point of any of the vaginal compartments protruding 1 cm or more below the level of the hymen was termed severe POP. These points are referred to as Ba, C or Bp ≥ 1cm in the POP-Q classification system.

**Table 1:** Stages of pelvic organ prolapse based on the POP-Q classification system.

Stage	Definition
0	No prolapse
I	The most distal portion of the prolapse is more than 1 cm above the level of the hymen
II	The most distal portion of the prolapse protrudes to 1 cm above—1 cm below the hymen
III	The most distal portion of the prolapse protrudes more than 1 cm below the hymen but does not form a complete prolapse
IV	Complete vaginal vault eversion or procidentia uteri (complete prolapse)

### Statistics

Data were coded and entered in Microsoft Access and exported to SPSS version 15.0 Inc, Chicago, IL, and cleaned by checking for duplication, missing values and outliers. Errors were corrected by referring to original questionnaires. Description of data was done using simple frequencies, mean and standard deviation. To determine risk factors associated with outcomes of interest (POP stage II–IV and severe POP), binary and multivariable logistic regression analyses were performed, having first defined POP stage II–IV as cases and those with stage 0–I as non-cases. All factors with P value < 0.05 in the bivariate logistic regression were entered into the multivariable model. Odds ratio (OR) with 95% confidence intervals (CI) were calculated. We then did the same for the second outcome of interest, severe POP, where cases were defined as POP with points Ba, C, Bp ≥ 1cm and non-cases were POP stage 0–I.

### Ethical considerations

Ethical clearance was obtained from the National Institute of Medical Research and the Ethical committee at Kilimanjaro Christian Medical University College. All participants gave verbal as well as a signed informed consent. A copy of the signed consent with information about the study and confidentiality of information remained with each of the participants. All women who presented at the clinic for pelvic examination were compensated for transportation costs ranging from 5000–15000 Tanzanian shillings depending on transport costs to the center. Women who needed further assessment and hospital treatment were referred to KCMC.

## Results

**Fig 1:**
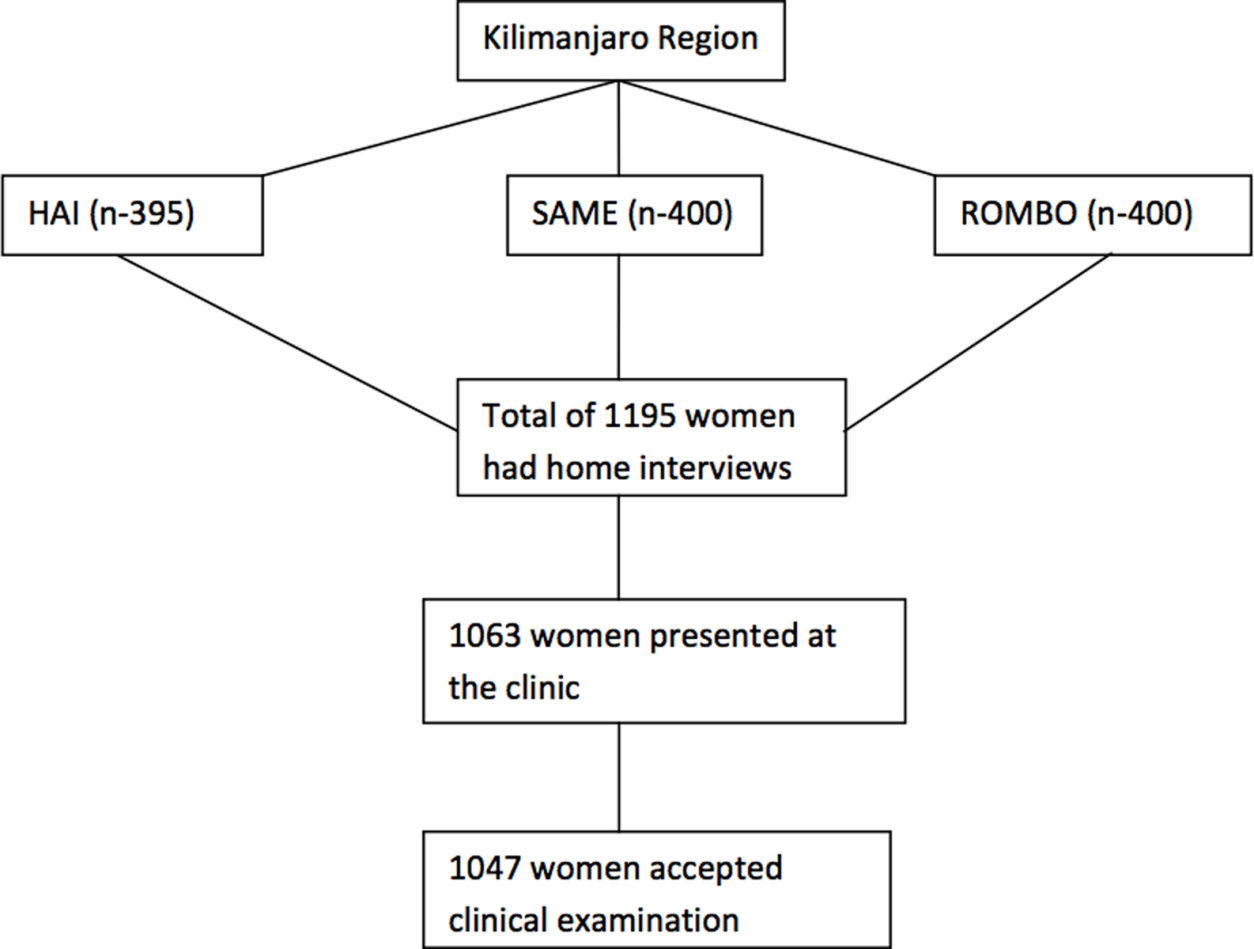
Study flowchart.

A total of 1195 female heads of households from the three districts were enrolled in the study, interviewed and invited for pelvic examination the next day. In all, 1063 (89%) women presented at the clinic of whom 1047 accepted a clinical examination (Fig 1). The median age of the participants was 46 years (range 18–90) and the median BMI was 25.5kg/m^2^ (range 15.2–49.3), while the majority of them, 828 (88.3%) had primary school education and 764 (73.0%) were farmers (Table 2). The median hours spent in heavy lifting were 2 hours per day (range 0–10).

**Table 2:** Socio-demographic and reproductive characteristics of participants.

Characteristics	n = 1047	%
***Age(years)***		
Median, range	46 (18–90)	
18–34	165	*15*.*8*
35–44	288	*27*.*5*
45–54	320	*30*.*6*
55–90	274	*26*.*2*
***Marital status***		
Married/cohabiting	816	*78*.*3*
Widowed	153	*14*.*7*
Single/separated	74	*7*.*1*
Missing	4	
***Educational level***		
No schooling	219	*20*,*9*
Primary school	706	*67*.*4*
Secondary school and above	122	*11*.*7*
***Occupation***		
Farmer	764	*73*.*0*
Business	261	*24*.*9*
Other	22	*2*.*1*
***Heavy lifting (hours per day)***		
Median, range	2 (0–10)	
0–1	408	*39*.*0*
2–4	578	*55*.*2*
5 +	61	*5*.*8*
***BMI categories (Kg/m2)***		
Median, range	25.5 (15.2–49.3)	
< 24	397	*38*.*0*
24.0–29.9	356	*34*.*1*
≥30	292	*27*.*9*
Missing	2	
***Parity***		
Median, range	5 (0–14)	
Para 0–1	193	*18*.*4*
Para 2–4	318	*30*.*4*
Para 5+	536	*51*.*2*
Missing	148	
***Age at first delivery (years)***		
Median, range	20 (14–40)	
10–19	347	*34*.*5*
20–29	627	*62*.*5*
30–40	30	*3*.*0*
Missing	43	
***Place of first delivery***		
At home	172	*16*.*4*
Prim. facility (dispensary/health centre)*	168	*16*.*0*
Hospital	707	*67*.*6*
***Mode of Delivery***		
Vaginal	970	*92*.*6*
Assisted	13	*1*.*2*
Caesarean section	65	*6*.*2*
***Duration of first labour (hours)***		
≤ 24	984	*93*.*9*
> 24	40	*3*.*9*
Don't recall	23	*2*.*2*
***Perenial tears***		
Yes	662	*63*.*2*
No	385	*36*.*8*

*In line with other Sub-Saharan African countries, Tanzania’s health system hierarchy starts from dispensary, health center, district, regional and finally, consultant hospitals. Primary health services are first offered by low-level providers at dispensaries and health centers with possibility of in-patient treatment at the latter

The median parity and age at first delivery were 5 children (range 0–14) and 20 years (range 14–40) respectively. For the first delivery, 707 women (67.6%) delivered in a hospital, and 970 women (92.6%) delivered vaginally, with a median birth weight of 3 kg (range 1–7). Forty women (3.9%) had been in labour for more than 24 hours and 63.2% sustained perineal tears (Table 2).

POP stage I, II, III and IV were demonstrated according to the POP-Q classification system in 302 (28.8%), 666 (63.6%), 6 (0.6%) and 4 (0.4%) women, respectively (Table 3). In sum, 64.6% of the women were found to have an anatomical POP stage of II–IV with the most predominant site being the anterior segment (62.7%), followed by the posterior segment (8.5%) and the central segment (1.8%).

**Table 3:** Prevalence of POP by anatomical site.

Anatomic Stage	Any segment n = 1047	Anterior segment n = 1047	Central segment n = 1047	Posterior segment n = 1047
Stage 0	69 (6.6%)	107 (10.2%)	568 (54.3%)	567 (54.2%)
Stage I	302 (28.8%)	284 (27.1%)	460 (43.9%)	391 (37.3%)
Stage II	666 (63.6%)	648 (61.9%)	16 (1.5%)	88 (8.4%)
Stage III	6 (0.6%)	5 (0.5%)	0 (0%)	1 (0.1%)
Stage IV	4 (0.4%)	3 (0.3%)	3 (0.3%)	0 (0%)

**Fig 2:**
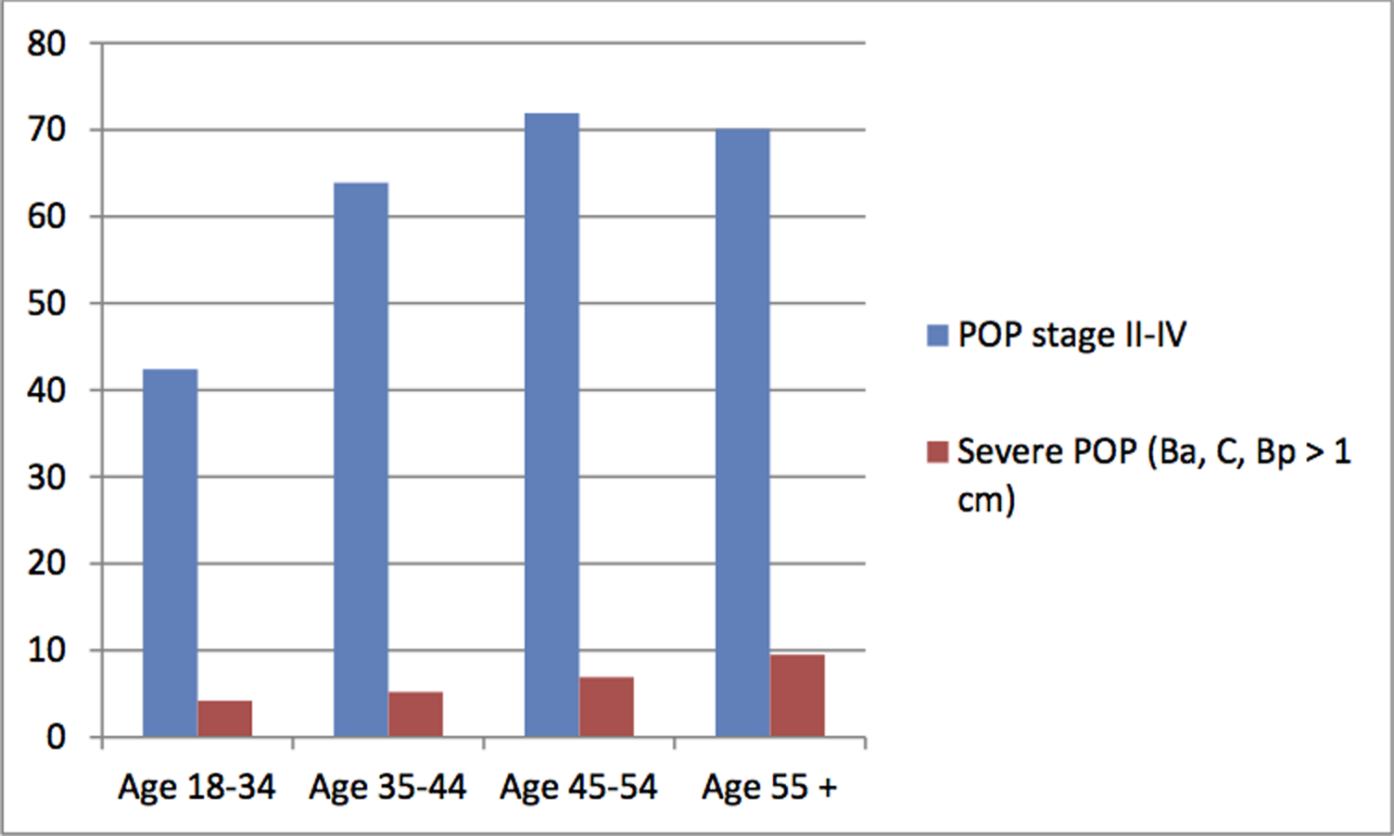
Frequency of POP stage II-IV and severe POP (Ba/C/Bp≥ 1cm) by age groups.

The prevalence of POP stage II–IV increased with advancing age: POP stage II–IV were found in 42.4%, 63.9%, 71.9% and 70.1% of women aged 18–34 years, 35–44 years, 45–54 years and 55 years and above, respectively (Fig 2). Of the 1047 women examined, 70 (6.7%) were found to have a severe POP which descended 1 cm or more below the hymen (Ba, C or Bp ≥ 1cm). In this group of women, there was a clear increasing prevalence rate across age groups. The occurrence of severe POP was especially pronounced among women aged 55 years and above, where one of out every ten women had a POP that descended 1 cm or more below the hymen.

Experiencing pelvic heaviness and feeling pressure in the lower abdomen were the most commonly reported symptoms followed by problems in emptying the bladder. These were reported by 16.1%, 12.4% and 10.6% of the women with POP stage II–IV, and 20.0%, 20.0% and 12.9% of the women with severe POP respectively. Among women with POP stage II–IV, 6.1% complained about ‘something falling out’ and 1.3% complained about ‘feeling something outside the vagina’. Respectively, these were reported by 10.0% and 12.9% of women with severe POP.

In the bivariate analyses, POP stage II–IV was significantly associated with high age, low education level (primary school and no schooling), being a farmer or doing petty trading, carrying heavy objects for ≥ 2 hours during daily routine work, high parity and having delivered at home or at a health centre when pregnant for the first time (Table 4). In the adjusted analyses, age of 35–44 (OR 1.62; 95%CI 1.07–2.47), age of 45–54 years (OR 1.87; 95%CI 1.21–2.90), being a farmer (OR 3.46; 95%CI 1.24–9.63) and doing petty trading (OR 2.89; 95% CI 1.02–8.14) were significantly associated with POP stage II–IV. Further, having delivered 3–4 times (OR 2.51; 95%CI 1.49–4.23) and 5 times or more (OR 6.10; 95% CI 3.48–10.7) were significantly associated with POP stage II–IV. Finally, women who had delivered at home or at a health centre in relation to their first delivery had an increased OR of 1.48 (95% CI 1.09–2.01) for having POP stage II–IV. But when adjusted for age and parity, being a farmer (OR 3.79; 95% CI 1.40–10.25) and petty trading (OR 3.22; 95% CI 1.17–8.86) were the only risk factors that remained significantly associated with POP stage II-IV.

**Table 4:** The association between socioeconomic characteristics, obstetric history and anatomic POP.

	Total:	Anatomical POP Stage II-IV vs Anatomical POP Stage 0-I			
	n = 1047	n = 676	OR (95% CI)	aOR (95% CI)§	aOR (95%CI) §§
***Age***					
18–34	165 (15.8%)	70 (10.3%)	1	1	-
35–44	288 (27.5%)	184 (27.2%)	**2.40(1.62–3.55)**	**1.62(1.07–2.47)**	**-**
45–54	320 (30.6%)	230 (34.0%)	**3.47(2.34–5.14)**	**1.87(1.21–2.90)**	**-**
55–90	274 (26.2%)	192 (28.4%)	**3.18(2.12–4.75)**	1.39(0.83–2.32)	-
***Education***					
No formal	219 (20.9%)	150 (22.2%)	**1.79(1.13–2.82)**	0.68(0.39–1.20)	0.90(0.53–1.53)
Primary	706 (67.4%)	459 (67.9%)	**1.53(1.03–2.25**)	0.87(0.56–1.36)	1.06(0.69–1.61)
Secondary+	122 (11.7%)	67 (9.9%)	1	1	1
***Occupation***					
Farmer	764 (73.0%)	522 (77.2%)	**5.75(2.22–14.9)**	**3.46(1.24–9.63)**	**3.79(1.40–10.25)**
Business	261 (24.9%)	148 (21.9%)	**3.49(1.32–9.21)**	**2.89(1.02–8.14)**	**3.22(1.17–8.86)**
Others	22 (2.1%)	6 (0.9%)	1	1	1
***Heavy work per day***					
0-1hr	408 (39.0%)	241 (35.7%)	1	1	1
2–4 hr	578 (55.2%)	391 (57.7%)	**1.44(1.11–1.87)**	1.20(0.90–1.61)	1.22(0.92–1.61)
5 + hr	61 (5.8%)	45 (6.7%)	**1.95(1.07–3.56)**	1.41(0.75–2.68)	1.39(0.75–2.61)
BMI*					
<-24	397 (38.0%)	260 (38.6%)	1	-	-
24–29	356 (34.1%)	230 (34.1%)	0.96(0.71–1.30)	-	-
30+	292 (27.9%)	182 (27.3%)	0.90(0.65–1.23)	-	-
***Parity***					
Para 0–2	193 (18.4%)	26 (3.8%)	1	1	-
Para 3–4	318 (30.4%)	237 (35.1%)	**2.12(1.48–3.06)**	**2.51(1.49–4.23)**	**-**
Para 5	536 (51.2%)	413 (61.1%)	**5.06(3.56–7.19)**	**6.10(3.48–10.7)**	**-**
Age first delivery**					
10–19	347 (34.6%)	237 (36.0%)	1	-	-
20–29	627 (62.5%)	405 (61.6%)	0.85(0.64–1.12)	-	-
30–40	30 (3.0%)	16 (2.4%)	0.53(0.25–1.13)	-	-
Place of first delivery***					
At home or health centre	339 (32.4%)	236(34.9%)	**1.39(1.05–1.83)**	**1.48 (1.09–2.01)**	1.21(0.83–1.75)
Hospital	707 (67.6%)	440 (65.1%)	1	-	1
Duration of first labour****					
< 24 hrs	984 (96.1%)	645 (96.6%)	1	-	-
≥ 24hrs	40 (3.9%)	23 (3.4%)	0.71(0.38–1.35)	-	-

*2 missing,

** 43 missing values,

*** 1 Missing value,

**** 23 missing values

§ Adjusted for age, education, occupation, parity, heavy work and place of first delivery

§§ Adjusted for age and parity

+secondary education and beyond

When focusing on severe POP, high age, being a farmer or doing petty trading, carrying heavy objects, high parity and having delivered at home or at a health centre when pregnant for the first time were associated with an increased risk of having severe POP (Table 5). After adjustment, carrying heavy objects for ≥ 5 hours (OR 4.70; 95% CI 1.67–13.2), having delivered 5 times or more (OR 10.2; 95% CI 2.22–48.6) and having delivered at home or at a health centre when pregnant for the first time (OR 2.40; 95% CI 1.36–4.22) were significantly associated with severe POP. And when adjusted for age and parity, carrying heavy objects for ≥ 5 hours (OR 3.5; 95%CI 1.47–8.36) was the only risk factor that remained significantly associated with severe POP.

**Table 5:** The association between socioeconomic characteristics, obstetric history and severe POP.

	Severe POP (Ba/C/Bp > 1 cm) vs Anatomical POP Stage 0-I			
	n = 70	OR (95% CI)	aOR (95% CI)§	aOR (95%CI) §§
***Age***				
18–34	7 (10%)	1	1	-
35–44	15 (21.4%)	1.96(0.77–5.01)	1.07(0.38–2.98)	-
45–54	22 (31.4%)	**3.32(1.35–8.14)**	1.28(0.46–3.60)	-
55–90	26 (37.1%)	**4.30(1.78–10.4)**	1.06(0.35–3.18)	-
***Education***				
No formal	17 (24.3%)	1.69(0.68–4.22)	-	-
Primary	45 (64.3%)	1.25(0.56–2.81)	-	-
Secondary+	8 (11.4%)	1	-	-
***Occupation***				
Farmer	56 (80.0%)	1.85(0.41–8.28)	-	-
Business	12 (17.1%)	0.85(0.17–4.15)	-	-
Others	2 (2.9%)	1	-	-
***Heavy work per day***				
0-1hr	18 (25.7%)	1	1	1
2–4 hr	43 (61.4%)	**2.12(1.18–3.82)**	1.79(0.95–3.38)	1.71(0.96–3.04)
5 + hr	9 (12.9%)	**5.22(2.02–13.5)**	**4.70(1.67–13.2)**	**3.5(1.47–8.36)**
BMI*				
<-24	35 (50%)	1	1	1
24–29	16 (22.9%)	**0.50(0.26–0.94)**	0.51(0.26–1.02)	**0.48(0.26–0.89)**
30+	19 (27.1%)	0.69(0.37–1.27)	0.81(0.42–1.60)	0.76(0.42–1.37)
***Parity***				
Para 0–2	2 (2.9%)	1	1	-
Para 3–4	18 (25.7%)	1.32(0.52–3.34)	2.55(0.55–11.8)	-
Para 5	50 (71.4%)	**5.89(2.68–13.0)**	**10.2(2.22–48.6)**	**-**
Age first delivery**				
10–19	27 (39.1%)	1	-	-
20–29	41 (59.4%)	0.75(0.44–1.29)	-	-
30–40	1 (1.4%)	0.29(0.04–2.31)	-	-
Place of first delivery***				
At home or health centre	34 (48.6%)	**2.45(1.45–4.12)**	**2.40(1.36–4.22)**	1.33(0.72–2.46)
Hospital	36 (51.4%)	1	1	1
Duration of first labour****				
< 24 hrs	69 (99.0%)	1	-	-
≥ 24hrs	1 (1.0%)	0.29(0.04–2.21)	-	-

* 2 missing,

** 43 missing values,

***1 missing value,

****23 missing values

§ Adjusted for Age, education, occupation, parity, heavy work and place of first delivery,

§§ Adjusted for age and parity,

+ secondary education and beyond

## Discussion

The prevalence of POP in this population-based study from Tanzania was 64.6% for POP stage II–IV and 6.7% for severe POP that descended 1 cm or more below the hymen. Having delivered 5 or more times was associated with a 10 times increased risk and delivery at home with a 2.5 times increased risk of severe POP. In addition, women carrying heavy objects had an almost five times increased risk of having severe POP.

In the current literature, the overall prevalence of POP varies from 3% to 56%, depending upon the definition utilized in establishing the POP diagnosis [3]. If the diagnosis is based on clinical evaluation the prevalence ranges from 41% to 56% as compared to 3% to 7% when the diagnosis is based on symptoms or complaints from women [3, 6, 18–20]. Our found prevalence is high compared to reported findings from other low-income countries such as Ethiopia (55%) [6] and Gambia (46%) [10]. This could be explained by the fact that our study population comprised rural Tanzanian women with a median age of 46 years, which is considerably higher than the reported mean age of 35 years in the Ethiopian study and 32 years in the Gambian study [6, 10]. Furthermore, in comparison with western countries the prevalence of POP in our study was higher than reported prevalence rates among African American women. In a study done in US the prevalence of POP was lower in African American women 1.9% as compared to white women 2.8% and Hispanic women 5.1% [21]. The difference in prevalence of POP between Africans residing in US and those living in Africa could be explained by a comparatively higher number of deliveries, difficult access to skilled delivery attendance and heavier physical work load among African living in sub-Saharan Africa.

There has been some discussion about how to define POP [11]. In our study population, 64.6% of the women had a POP stage II–IV. It may be argued that it makes little sense to define a benign condition which is that common as a clinically relevant problem. We therefore applied a more restricted approach and defined women as having a severe POP, if the prolapse descended 1 cm or more below the hymen, since we believe that women having a POP descending at this level would be bothered by symptoms and thus in need of treatment. Based on this definition, 6.7% of the women had severe POP. Our findings are in accordance with a population based study from Ethiopia, where 7.2% and of the women were found to have a clinically relevant prolapse [6] while another population based study from Gambia reported that 14.7% of the included women had a POP that was severe enough to warrant surgical intervention [4].

We found a low prevalence of reported symptoms with only 6.1% in the group of women with POP stage II–IV complaining about a feeling of something falling out of the vagina. The same applied for 18.6% of women with severe POP. Other studies from low-income settings have similarly found a poor correlation between an objective POP on clinical examination and reported prolapse symptoms [4]. The low prevalence of symptoms may reflect under-reporting. We suspect that although the research assistants were qualified, trained nurses, the women might have regarded the questions focusing on POP symptoms as somewhat intrusive and thus been reluctant to answer them. To get a more trustworthy picture of Tanzanian women’s symptoms in relation to POP, steps should be taken to increase and ensure the validity of the POPDI-6, so it can be applied in a Tanzanian context.

We found an association between increasing age and POP stage II–IV. Multiple population based studies have clearly demonstrated a similar association [3, 5, 12]. For instance, an American study found that the proportion of women having symptomatic POP increased from 1.6% in women aged 20–39 to 4.1% in women aged 80 years or older [21]. Likewise, a Gambian study found a strong association between POP and advancing age where women aged 45–54 years had a two times higher risk of POP compared to women aged 15 to 24 years [4]. The relationship between POP and advancing maternal age is attributed to the decline in available estrogen after menopause and the associated changes in the composition of the connective tissue [22].

We also found a strong association between increasing parity and POP, where women who had delivered 5 times or more had a 6 times increased risk of POP stage II–IV and a 10 times increased risk of severe POP. Our findings are in accordance with the Gambian study where women with eight or more deliveries had a 15 times higher risk of POP [4]. This reflects that excessive stretching, tearing and multiple deliveries are the main predisposing obstetric factor for developing POP [3]. Having delivered at home or at a health centre when pregnant for the first time was another obstetric risk factor associated with an increased risk of POP, implying that home delivery in a low-income setting with poor infrastructure is linked with an increased risk of prolonged labour. This is consistent with other studies from low-income countries where it has been documented that women who have experienced a prolonged labour have an almost two times increased risk of POP II–IV [3, 6].

Farming women and women who were involved in petty trading had an increased risk of POP stage II–IV. This finding reflects that women involved in such occupations often carry heavy objects in relation to their working activities. The same association was not found among women with severe POP, most likely because the effect of occupation was outweighed by the effect of heavy work. Hence, women who carried out heavy work for five hours or more daily had an almost five times increased risk of severe POP. Similar findings have been reported from Ethiopia and Nepal [6, 12]. It’s interesting to note that our results are not in agreement with previous research findings, which have demonstrated the association between BMI and POP. A recent systematic review and meta-analysis have shown that obese and overweight women are more likely to develop POP compared to women with normal BMI [23]. The observed difference could be explained by a selection bias, as only one out of 22 eligible studies included in the meta-analyses was conducted in an African setting. Most of the studies were conducted in Europe and America with estimated high prevalence of obesity and overweight. Furthermore, in a Tanzanian context obese women are often wealthier and less likely to carry out heavy work. Thus among obese women, the effect of obesity may have been masked by the effect of less exposure to heavy work.

The main strength of our study is that it is based on a large sample size where women were selected through cluster randomization. In addition, the number of women who accepted clinical examination was remarkably high. The results are thus likely to be representative for the general population of women in Kilimanjaro Region, Tanzania. In addition, the clinical examinations were performed by two experienced gynaecologists and a resident who received thorough training in using the POP-Q classification system. There are, however, also some limitations of the study. We included women who were head of households and this may have resulted in an underrepresentation of younger women. Further, the study may be hampered by its failure to apply a culturally validated tool to assess POP symptoms.

In conclusion, this study highlights that POP is a common condition among rural Tanzanian women where 64.6% are having a POP grade II-IV and 6.7% are having a severe POP descending 1 cm or more below the hymen. Increasing age, many vaginal deliveries, unskilled delivery attendants and frequent heavy lifting is associated with a significant increased risk of POP among Tanzanian women. With an ageing population in Tanzania as well as in other low-income countries, we suggest that more attention is given to address the profound consequences of POP, especially among elderly women living in rural areas.

## Supporting information

S1 Dataset POP prevalence in rural Kilimanjaro_PLOS ONE.sav. (SAV)
